# Effects of acute bouts of endurance exercise on retinal vessel diameters are age and intensity dependent

**DOI:** 10.1007/s11357-014-9650-3

**Published:** 2014-04-12

**Authors:** M. Nussbaumer, L. Donath, M. Fischer, J. Schäfer, O. Faude, L. Zahner, A. Schmidt-Trucksäss, H. Hanssen

**Affiliations:** 1 Department of Sport, Exercise and Health, Division of Sports and Exercise Medicine, Medical Faculty, University of Basel, Birsstrasse 320B, 4052 Basel, Switzerland; 2 Department of Sport, Exercise and Health, Division of Movement and Exercise Science, Medical Faculty, University of Basel, Basel, Switzerland; 3 Basel Institute for Clinical Epidemiology and Biostatistics, University Hospital Basel, Basel, Switzerland

**Keywords:** Endurance exercise, Retinal vessel diameters, Microvascular reactivity, Vascular ageing

## Abstract

Alterations of retinal vessel diameters are associated with increased cardiovascular risk. We aimed to investigate changes in retinal vessel diameters in response to acute dynamic exercise of different intensities and whether these changes are age dependent. Seventeen healthy seniors (median (IQR) age 68 (65, 69) years) and 15 healthy young adults (median (IQR) age 26 (25, 28) years) first performed a maximal treadmill test (MTT) followed by a submaximal treadmill test (SMTT) and a resting control condition in randomised order. Central retinal arteriolar (CRAE) and central retinal venular (CRVE) diameter equivalents were measured before as well as 5 (t_5_) and 40 (t_40_) minutes after exercise cessation using a static retinal vessel analyser. Both exercise intensities induced a significant dilatation in CRAE and CRVE at t_5_ compared to the control condition (*P* < 0.001). At t_40_, the mean increase in CRAE and CRVE was greater for MTT compared to that for SMTT (CRAE 1.7 μm (95 % confidence interval (CI) −0.1, 3.6; *P* = 0.061); CRVE 2.2 μm (95 % CI 0.4, 4.1; *P* = 0.019)). However, the estimated difference at t_5_ between seniors and young adults in their response to MTT compared to SMTT was 5.3 μm (95 % CI 2.0, 8.5; *P* = 0.002) for CRAE and 4.1 μm (95 % CI −0.4, 8.6; *P* = 0.076) for CRVE. Wider arteries and veins after maximal versus submaximal exercise for seniors compared to young adults suggest that myogenic vasoconstriction in response to exhaustive exercise may be reduced in seniors. Age-related loss of vascular reactivity has clinical implications since the arteriolar vasoconstriction protects the retinal capillary bed from intraluminal pressure peaks.

**Electronic supplementary material:**

The online version of this article (doi:10.1007/s11357-014-9650-3) contains supplementary material, which is available to authorized users.

## Introduction

In recent years, great emphasis has been placed on the identification of tissue biomarkers to determine vascular dysfunction early in the process of atherosclerosis. Retinal vessel diameters are a new tissue biomarker for the assessment of cardiovascular risk in all age groups and are affected early in the process of cardiovascular disease (Wong et al. 2002; Tedeschi-Reiner et al. 2005; Wong et al. 2006a). Retinal vessels are an easily accessible part of the microcirculation and share common functional, morphological and embryological characteristics with the cerebrovascular bed (Netter 2006). Large cohort studies have previously shown that narrower retinal arterioles, wider retinal venules and a lower arteriolar-to-venular diameter ratio (AVR) are associated with increased risk and severity of hypertension (Wang et al. 2003; Ikram et al. 2006b; Kawasaki et al. 2009), increased risk of stroke (Ikram et al. 2006a; McGeechan et al. 2009) and higher cardiovascular morbidity and mortality (Wong et al. 2002; Wang et al. 2006a; Wang et al. 2006b; Wang et al. 2007). In elderly persons, smaller retinal arteriolar diameters are associated with incident coronary heart disease and larger retinal venular diameters are independently associated with increased risk of cardiovascular disease (Wong et al. 2006b).

It is therefore of high clinical relevance and interest to examine how intervention strategies, such as dynamic exercise, affect retinal vessel diameters. To date, little is known about the chronic and acute effects of endurance exercise on retinal vessel diameters. In large population-based cross-sectional studies, lower levels of physical activity are associated with wider retinal venules (Tikellis et al. 2010; Anuradha et al. 2011). In the only available chronic exercise intervention study, we have previously shown that higher physical fitness levels are associated with higher retinal AVR and that regular endurance exercise induces arteriolar dilatation and venular constriction in middle-aged lean and obese individuals (Hanssen et al. 2011).

Progressive exercise is known to lower intraocular pressure and raise retinal arterial pressure. In response to the increase in perfusion pressure, a vasoconstriction of retinal arteries and veins occurs, inducing an increase in vascular resistance. This compensatory autoregulation, also known as myogenic response, is responsible for the maintenance of normal blood flow in central retinal arteries and veins during dynamic exercise (Harris et al. 1996; Iester et al. 2007; Hayashi et al. 2011). In seniors, the retinal arterial myogenic response induced by a systemic blood pressure increase during isometric resistance training has been shown to be decreased compared to younger adults (Jeppesen et al. 2004).

The physiological regulation of the retinal microcirculation during exercise is complex. The above literature suggests that exercise intensity, ventilatory conditions, age and time of measurement have to be taken into consideration when assessing retinal vessel diameters in response to acute bouts of exercise. The interest in retinal vessel diameters is based upon the fact that they are regulators of local blood flow, valid and robust microvascular surrogate biomarkers of cardiovascular risk and can be analysed very easily. In our study, we therefore aimed to investigate retinal vessel diameters after submaximal (walking-based activity) and maximal (exhaustion) endurance exercise in healthy young adults and seniors. We hypothesized that the myogenic autoregulation of retinal vessels and the corresponding vasoconstriction is enhanced after maximal endurance exercise and may be reduced at older age.

## Methods

### Study participants

Volunteers were prospectively recruited from the local community. Inclusion criteria were an age of 18–40 years in the group of young adults and of 60–80 years in the group of seniors. Exclusion criteria were the presence of cardiovascular, metabolic or pulmonary disease or limiting orthopaedic problems. The criteria were assessed by taking the medical history, using the physical activity readiness questionnaire (PAR-Q) (Armstrong et al. 2006), performing a physical cardiopulmonary examination, taking the blood pressure at rest and by completing an electrocardiogram (ECG) during rest and maximal exercise. The study was approved by the local ethics committee (Ethikkommission beider Basel, Switzerland). Written informed consent was obtained from all study participants.

### Study design

**Fig. 1:**
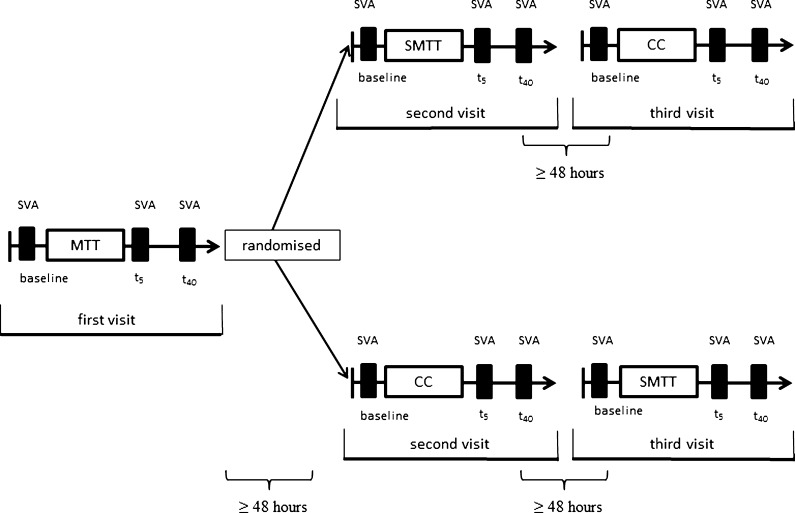
Participants were examined in a cross-over design. At the first visit, all study participants performed a maximal treadmill test (*MTT*). At the second and third visits, participants performed a submaximal 2-km treadmill test (*SMTT*) and a control condition (*CC*) in randomised order. Central retinal arteriolar (*CRAE* (in micrometre)) and venular (*CRVE* (in micrometre)) equivalents were measured before and after exercise cessation using a static retinal vessel analyser (*SVA*)

Participants were examined in a cross-over design at three visits, which were at least 48 h apart (Fig. 1). All three visits were intraindividually performed at the same time of day to avoid diurnal variations. At the first visit, all study participants performed a maximum treadmill test (MTT). At the second and third visits, participants performed a submaximal 2-km treadmill test (SMTT) and a resting control condition (CC) in randomised order. The intensity of the SMTT was determined based on the ratings of perceived exertion during the MTT. Therefore, the MTT had to be implemented at the first visit for all study participants. We measured central retinal arteriolar equivalent (CRAE) and central retinal venular equivalent (CRVE) before as well as 5 (t_5_) and 40 (t_40_) minutes after exercise cessation using a static retinal vessel analyser (SVA-T). The first postexercise measurement was fixed at 5 min after cessation of the exercise bout as the earliest time point to ensure standardization after spiroergometry and to prevent orthostatic dysregulation during measurements. Participants were tested in the postprandial state (2 h) and were instructed to refrain from caffeine and alcoholic beverages for 12 h and avoid exercise for 24 h prior to testing.

### Static retinal vessel analysis

CRAE and CRVE were the primary outcomes in this exploratory study. Each parameter was measured using the static retinal vessel analyser (SVA-T, Imedos Systems UG, Jena, Germany). The system consists of a fundus camera and analysing software, allowing non-invasive and non-mydriatic assessment of retinal vessel diameters. Two valid images from the retina of the left and right eyes, with an angle of 30° and with the optic disc in the centre, were captured per time point. Retinal arterioles and venules, coursing through an area of 0.5–1 disc diameter from the optic disc margin, were identified using special analysing software (Vesselmap 2, Visualis, Imedos Systems UG). Vessel diameters ≤45 μm were generally discarded. Diameters were averaged to CRAE and CRVE, using the Parr-Hubbard formula described elsewhere (Hubbard et al. 1999). CRAE and CRVE are generally presented in measuring units (mu). In the model of Gullstrand’s normal eye, 1 mu relates to 1 μm. Reliability of this method has been shown to be high, with interobserver and intraobserver interclass correlation coefficients for arteriolar and venular diameter measurements ranging from 0.78 to 0.99 (Hubbard et al. 1999; Wong et al. 2004; Wang et al. 2008).

### Protocol of exercise modes and control condition

#### Maximum treadmill test (MTT)

The walking-based protocol started at a velocity of 1.5 miles (seniors) or 3.5 miles (young adults) per hour with an inclination of 0 %. Intensity was increased every minute by increasing either the inclination or the velocity until volitional exhaustion was reached (Peterson et al. 2003).

#### Submaximal 2-km treadmill test (SMTT)

Participants had to walk a distance of 2 km at an individually determined submaximal intensity on the treadmill. The velocity and inclination reached at the CR-10 Borg value of 4 during the MTT were set as the intensity for SMTT, a comfortable walking-based submaximal exercise intensity (Oja et al. 2008; Donath et al. 2013).

#### Resting control condition (CC)

Participants had to rest in a supine position in a dimly lit room with no distractions for 30 min.

During all three MTT, SMTT and CC, relative peak oxygen consumption (VO_2peak_), respiratory exchange ratio (RER), partial pressure of end-tidal carbon dioxide (PetCO_2_), minute ventilation/respiratory minute volume (V’E), tidal volume (VT), maximum heart rate (HR_max_) and rate of perceived exertion (Borg-scale, CR-10) were recorded. Ventilatory parameters were obtained using the breath-by-breath Cortex Metalyzer^®^ 3B metabolic test system (Cortex Biophysik GmbH, Leipzig, Germany), and HR was measured using the Custo cardio 100 12-channel PC ECG system (Custo med GmbH, Ottobrunn, Germany). Partial pressure of carbon dioxide in the blood (PaCO_2_) was calculated from PetCO_2_ as described before (Jones et al. 1979).

### Statistical analyses

We used baseline-adjusted fixed effects models to compare retinal vessel diameters after MTT, SMTT and CC. In our models, we included an exercise mode effect, a time effect and an interaction effect between exercise mode and time to indicate that differences in retinal vessel diameter in response to exercise may differ at t_5_ and t_40_. Baseline retinal vessel diameter levels were included as a covariate in our models. For the main analysis, we considered the participant effect being fitted as fixed. We also report results of two sensitivity analyses. First, we used baseline-adjusted random effects models to compare retinal vessel diameters after MTT, SMTT and CC, with the participant effect being fitted considered as random. Second, we just considered the SMTT and CC periods of a participant in order to be able to additionally adjust for the potentially confounding effect of period. We used residual plots of scaled residuals to detect any outliers or a general lack of normality.

A key question for this study was whether seniors and young adults differ in the way in which they respond to exercise. To address this question, we compared the expected difference in retinal vessel diameter between two exercise modes between seniors and young adults while adjusting for baseline differences in retinal vessel diameter between the two exercise modes.

For each analysis, we report an estimate (with 95 % confidence interval) of the difference between two exercise modes and of the difference in the way in which seniors and young adults respond to exercise. We used SAS version 9.2 (SAS Institute Inc., Cary, NC, USA) for our analyses; for graphics, we used R version 3.0.1 (R Foundation for Statistical Computing, Vienna, Austria) and the R add-on package *lattice* version 0.20–23 (Sarkar 2008).

## Results

### Participant characteristics

From a total of 43 volunteers recruited from the local community, 17 healthy seniors and 15 healthy young adults were included in the study. Eleven seniors were excluded due to antihypertensive or diabetic medication (*n* = 3), macular degeneration or poor quality of retinal images (*n* = 7) or failure to perform the study protocol (*n* = 1). Included seniors were more likely to be female and to have higher systolic and diastolic blood pressure than young adults (Table 1).

**Table 1:** Participant characteristics with continuous data summarised as median (interquartile range) and categorical data as *n* (%)

Characteristic	All participants (*n* = 32)	Seniors (*n* = 17)	Young adults (*n* = 15)
Age, years		68 (65, 69)	26 (25, 28)
Female gender	19 (59)	12 (71)	7 (47)
Height, cm	168 (164, 178)	167 (163, 170)	175 (167, 181)
Weight, kg	66 (58, 76)	66 (62, 75)	67 (57, 77)
BMI, kg/m^2^	23 (22, 25)	24 (22, 26)	22 (19, 23)
Baseline SBP^a^, mmHg	124 (115, 137)	133 (124, 139)	115 (108, 124)
Baseline DBP^a^, mmHg	80 (74, 88)	85 (80, 88)	78 (73, 81)
Baseline AVR	0.87 (0.83, 0.9)	0.87 (0.83, 0.91)	0.87 (0.84, 0.9)
Baseline CRAE, in μm	188 (177, 194)	188 (176, 194)	190 (180, 194)
Baseline CRVE, in μm	216 (203, 229)	209 (203, 234)	224 (210, 228)

Baseline measurements are those taken at the first visit before the start of maximal exercise testing

*BMI* body mass index, *SBP* systolic blood pressure, *DBP* diastolic blood pressure, *AVR* arteriolar-to venular diameter ratio, *CRAE* central retinal arteriolar equivalent, *CRVE* central retinal venular equivalent

^a^Available in 14 (82 %) and 15 (100 %) seniors and young adults, respectively

Of the 32 study participants, 13 (41 %; eight seniors and five young adults) and 19 (59 %; nine seniors and 10 young adults) participants were randomly allocated to the exercise mode sequences MTT-SMTT-CC and MTT-CC-SMTT, respectively.

### Exercise performance and control condition

Study participants showed good endurance capacity according to the peak oxygen consumption (Table 2). Seniors and young adults performed the SMTT at a median (interquartile range) intensity of 74 % (70 % 79 %) and 78 % (70 %, 84 %) of the maximum heart rate (HR_max_), respectively.

**Table 2:** Exercise performance and control condition with data summarised as median (interquartile range)

Performance measure	All participants (*n* = 32)	Seniors (*n* = 17)	Young adults (*n* = 15)
Maximum treadmill test (MTT)			
Relative VO_2peak_^a^, mL/min/kg	38 (31, 51)	31 (25, 34)	52 (45, 56)
HR_max_^b^, bpm	172 (160, 192)	160 (157, 167)	193 (184, 195)
RER^a^	1.16 (1.09, 1.21)	1.1 (1.03, 1.18)	1.18 (1.14, 1.21)
PetCO_2_^d^, mmHg	34.0 (32.2, 37.6)	33.2 (31.1 , 34.4)	36.3 (33.2, 39.0)
PaCO_2_^d^, mmHg	36.1 (34.5, 39.3)	35.3 (33.5, 36.5)	38.2 (35.4, 40.6)
V’E^d^, L/min	91 (67, 136)	57 (56, 90)	129 (84, 149)
VT^d^, L	2.2 (1.8, 2.8)	1.6 (1.5, 2.3)	2.6 (2.0, 3.4)
Submaximal 2-km treadmill test (SMTT)			
Relative VO_2_, mL/min/kg	26 (19, 30)	19 (17, 23)	30 (28, 32)
HR, bpm	127 (122, 143)	122 (112, 126)	142 (135, 162)
RER	0.93 (0.88, 1.0)	0.89 (0.82, 0.94)	0.97 (0.93, 1.01)
PetCO_2_^d^, mmHg	37.1 (36.1, 40.1)	36.5 (34.4, 37.0)	39.0 (36.7, 41.7)
PaCO_2_^d^, mmHg	38.9 (38.0, 41.6)	38.4 (36.4, 38.8)	40.6 (38.6, 43.1)
V’E^d^, L/min	48 (39, 57)	33 (30, 39)	55 (48, 60)
VT^d^, L	1.7 (1.3, 1.9)	1.2 (1.1, 1.6)	1.9 (1.5, 2.2)
Control condition (CC)			
Relative VO_2_^c^, mL/min/kg	3.5 (3, 4)	3 (2.8, 4)	3.6 (3.5, 4)
HR^d^, bpm	56 (54, 65)	64 (53, 68)	55 (54, 59)
RER^c^	0.86 (0.82, 0.9)	0.83 (0.82, 0.9)	0.87 (0.85, 0.9)
PetCO_2_^d^, mmHg	36.2 (32.8, 38.0)	34.3 (31.5, 36.4)	36.8 (34.7, 39.0)
PaCO_2_^d^, mmHg	38.1 (35.1, 39.7)	36.4 (33.9, 38.3)	38.6 ( 36.7, 40.6)
V’E^d^, L/min	8 (6, 9)	7 (6, 8)	8 (8, 9)
VT^d^, L	0.5 (0.4, 0.6)	0.5 (0.4, 0.5)	0.6 (0.5, 0.7)

*VO*_*2peak*_ peak oxygen consumption, *VO*_*2*_ oxygen consumption, *HR*_*max*_ maximum heart rate, *HR* heart rate, *bpm* beats per minute, *RER* respiratory exchange ratio, *PetCO*_*2*_ partial pressure of end-tidal carbon dioxide, *PaCO*_*2*_ estimated arterial partial pressure of carbon dioxide, *V’E* minute ventilation/respiratory minute volume, *VT* tidal volume

^a^Available in 15 (88 %) and 15 (100 %) seniors and young adults, respectively

^b^Available in 17 (100 %) and 14 (93 %) seniors and young adults, respectively

^c^Available in 16 (94 %) and 15 (100 %) seniors and young adults, respectively

^d^Available in 8 (47 %) and 15 (100 %) seniors and young adults, respectively

### Retinal vessel diameters in response to exercise

**Fig. 2:**
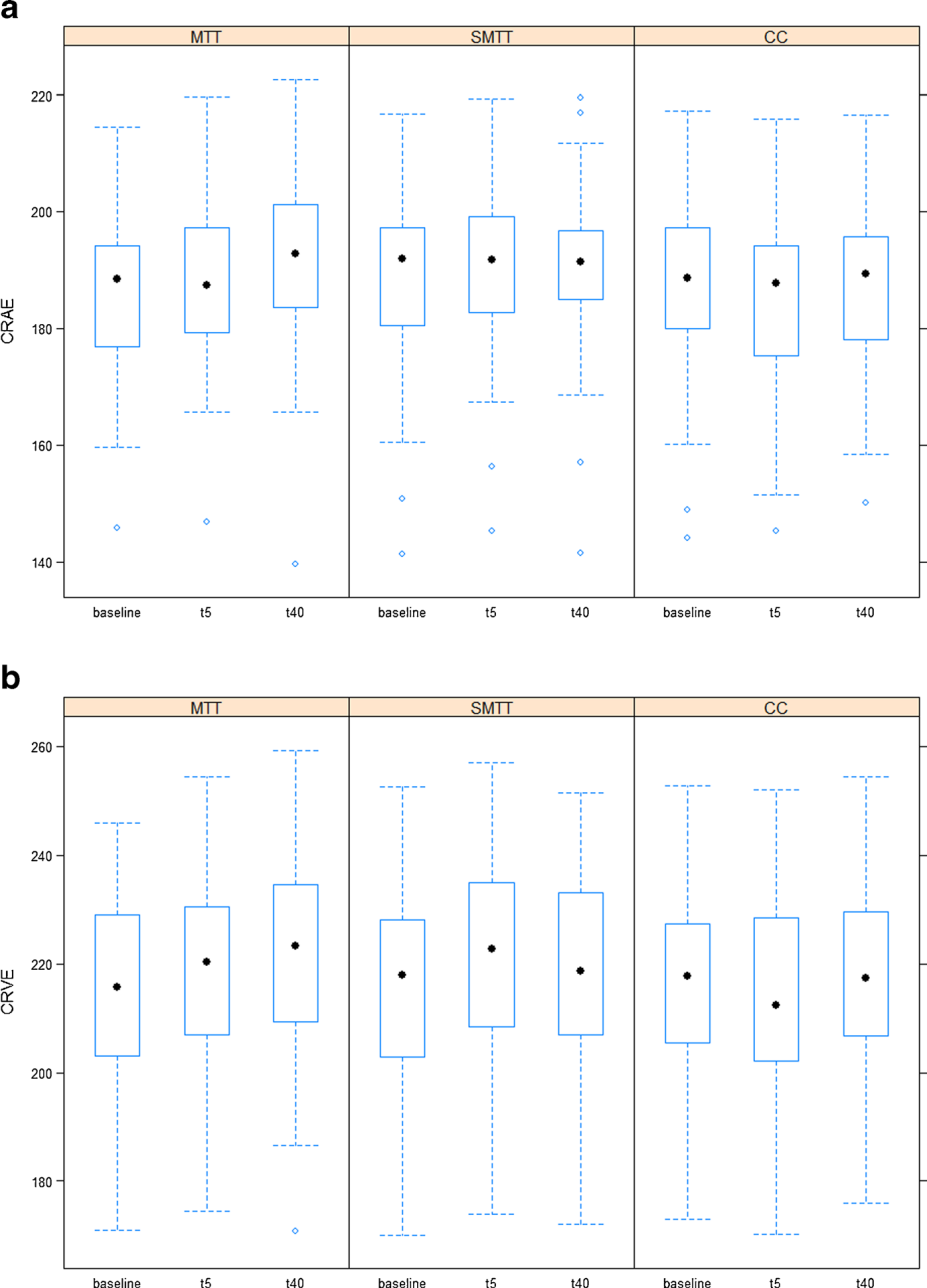
Retinal vessel diameters at baseline as well as 5 min (*t*_5_) and 40 min (*t*_40_) after a maximal treadmill test (*MTT*), a submaximal 2-km treadmill test (*SMTT*) and a resting control condition (*CC*). **a** Central retinal arteriolar equivalent (*CRAE* (in micrometre)). **b** Central retinal venular equivalent (CRVE (in micrometre))

MTT induced a continuous dilatation from baseline to t_5_ to t_40_ in both CRAE and CRVE (Fig. 2a–b). For SMTT, both CRAE and CRVE dilated from baseline to t_5_, followed by no further dilatation at t_40_. For CC, the narrowest CRAE and CRVE were observed at t_5_, with slightly wider CRAE and CRVE both at baseline and at t_40_. One participant did not have measurements of CRAE and CRVE at t_40_ for CC.

### Effects of exercise mode on retinal vessel diameters

Both MTT and SMTT led to a significant dilatation in CRAE and CRVE relative to CC, with estimated differences between 3.3 and 6.3 μm (Table 3). At t_5_, the dilatation in CRAE and CRVE was slightly less pronounced for MTT compared with SMTT (CRAE −1.2 μm (95 % confidence interval (CI) −3.0, 0.7; *P* = 0.206); CRVE: −1.1 μm (95 % CI −3.0, 0.7; *P* = 0.224)). At t_40_, the increase in CRAE and CRVE was greater for MTT compared with SMTT (CRAE 1.7 μm (95 % CI −0.1, 3.6; *P* = 0.061); CRVE: 2.2 μm (95 % CI 0.4, 4.1; *P* = 0.019)). The graphs of the scaled residuals indicated no appreciable departure from normality nor any definite outlying observations.

**Table 3:** Estimated baseline-adjusted exercise mode effects on mean central retinal arteriolar equivalent (CRAE) and on mean central retinal venular equivalent (CRVE)

		Estimate (95 % CI)	*P* value
CRAE, μm			
t_5_ exercise mode effects	SMTT–CC	4.9 (3.1, 6.7)	<0.001
	MTT–CC	3.7 (1.9, 5.5)	<0.001
	MTT–SMTT	−1.2 (−3.0, 0.7)	0.206
t_40_ exercise mode effects	SMTT–CC	3.3 (1.5, 5.2)	<0.001
	MTT–CC	5.1 (3.2, 6.9)	<0.001
	MTT–SMTT	1.7 (−0.1, 3.6)	0.061
CRVE, μm			
t_5_ exercise mode effects	SMTT–CC	6.3 (4.4, 8.1)	<0.001
	MTT–CC	5.1 (3.3, 7.0)	<0.001
	MTT–SMTT	−1.1 (−3.0, 0.7)	0.224
t_40_ exercise mode effects	SMTT–CC	3.7 (1.8, 5.5)	<0.001
	MTT–CC	5.9 (4.0, 7.7)	<0.001
	MTT–SMTT	2.2 (0.4, 4.1)	0.019

*CI* confidence interval, *CRAE* central retinal arteriolar equivalent, *CRVE* central retinal venular equivalent, *MTT* maximal treadmill test, *SMTT* submaximal treadmill test, *CC* control condition

### Sensitivity analyses

Random effects models led to a similar pattern of estimated differences in retinal vessel diameter between the exercise modes, with differences apparent for MTT and SMTT, both relative to CC, and for MTT relative to SMTT only at t_40_ (data not shown).

Estimated differences between SMTT and CC appear robust to possible confounding by period. When adjusting for a period effect, the estimated difference in CRAE between SMTT and CC was 5.1 μm (95 % CI 3.3, 6.9; *P* < 0.001) and 3.5 μm (95 % CI 1.7, 5.3; *P* < 0.001) at t_5_ and t_40_, respectively, and the estimated difference in CRVE was 6.4 μm (95 % CI 4.6, 8.1; *P* < 0.001) and 3.7 (95 % CI 1.9, 5.5; *P* < 0.001) at t_5_ and t_40_, respectively.

### Effects of age on mean difference in retinal vessel diameters

**Fig. 3:**
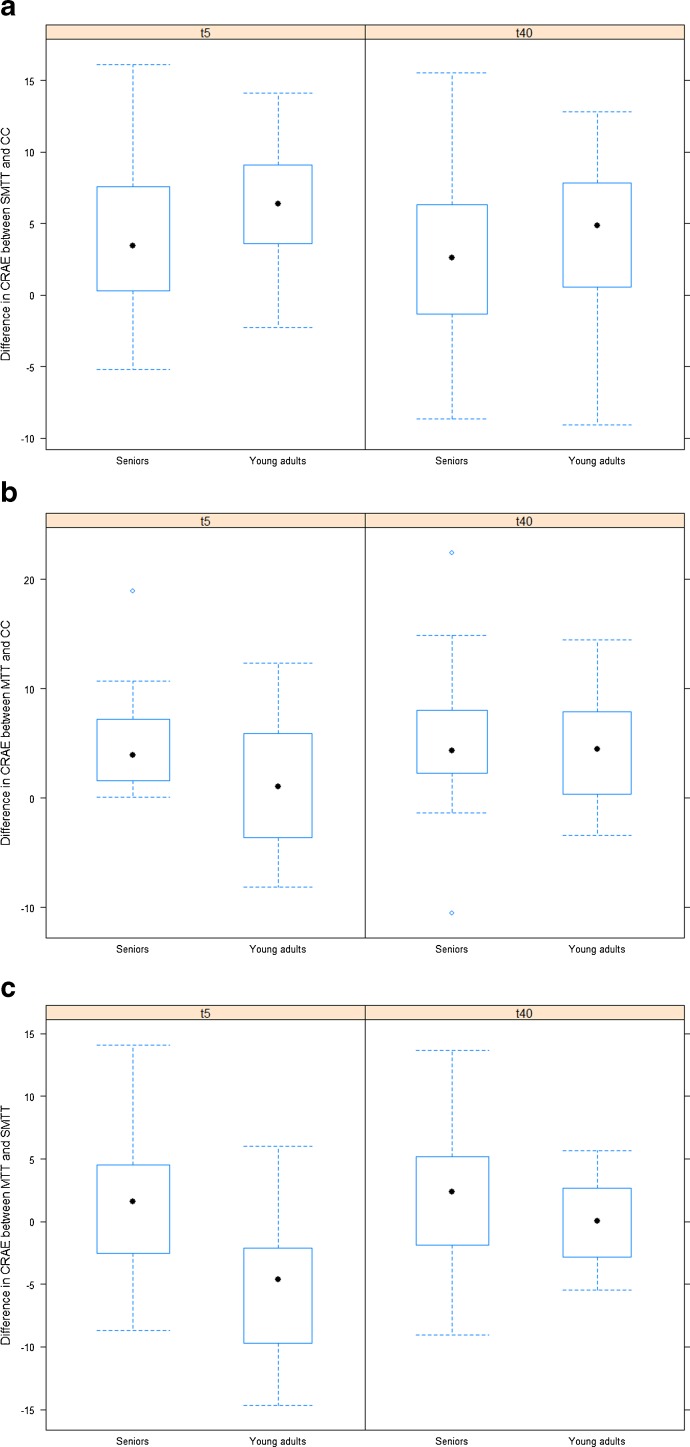

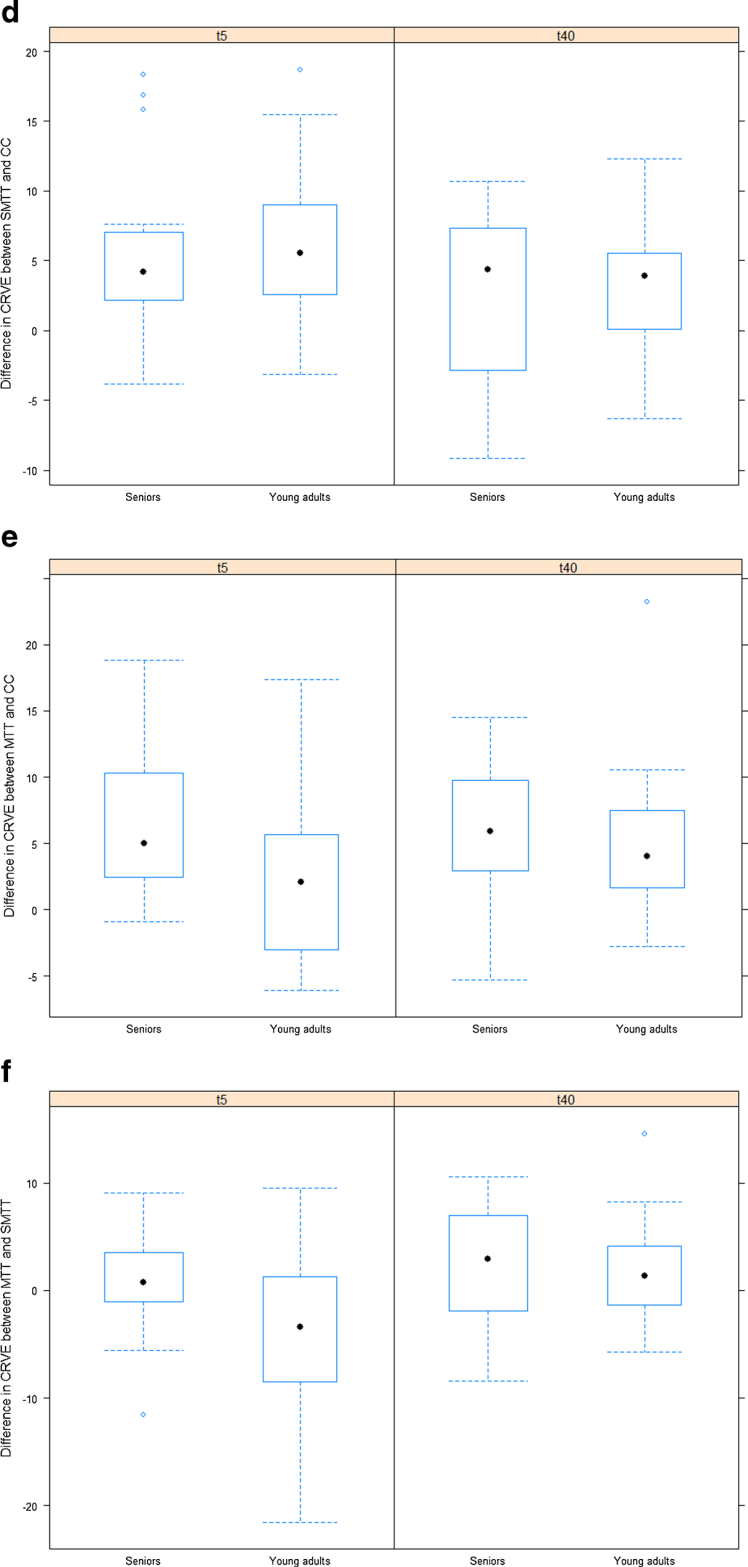
Differences in retinal vessel diameter between maximal treadmill test (*MTT*), submaximal 2-km treadmill test (*SMTT*) and resting control condition (*CC*) for seniors and for young adults. **a** Difference in central retinal arteriolar equivalent (*CRAE* (in micrometre)) between *SMTT* and *CC* for seniors and for young adults. **b** Difference in *CRAE* between *MTT* and *CC* for seniors and for young adults. **c** Difference in *CRAE* between *MTT* and *SMTT* for seniors and for young adults. **d** Difference in central retinal venular equivalent (*CRVE* (in micrometre)) between *SMTT* and *CC* for seniors and for young adults. **e** Difference in *CRVE* between *MTT* and *CC* for seniors and for young adults. **f** Difference in *CRVE* between *MTT* and *SMTT* for seniors and for young adults

The dilatation in CRAE and CRVE after SMTT relative to CC was less pronounced in seniors compared with young adults at t_5_ and t_40_ (Fig. 3a–f; Table 4).

**Table 4:** Estimated baseline-adjusted age-group effects (comparing seniors with young adults) on mean difference in central retinal arteriolar equivalent (CRAE) and central retinal venular equivalent (CRVE) between each two exercise modes

		Estimate (95 % CI)	*P* value
CRAE, μm			
t_5_ difference in exercise response between seniors and young adults	SMTT–CC	−2.4 (−6.2, 1.3)	0.190
	MTT–CC	2.9 (−0.5, 6.3)	0.088
	MTT–SMTT	5.3 (2.0, 8.5)	0.002
t_40_ difference in exercise response between seniors and young adults	SMTT–CC^a^	−1.7 (−6.1, 2.7)	0.439
	MTT–CC^a^	0.3 (−4.2, 4.9)	0.878
	MTT–SMTT	1.4 (−2.3, 5.1)	0.438
CRVE, μm			
t_5_ difference in exercise response between seniors and young	SMTT–CC	0.4 (−4.1, 5.0)	0.848
	MTT–CC	5.0 (1.5, 8.4)	0.006
	MTT–SMTT	4.1 (−0.4, 8.6)	0.076
t_40_ difference in exercise response between seniors and young	SMTT–CC^a^	1.0 (−2.7, 4.8)	0.572
	MTT–CC^a^	1.7 (−2.3, 5.6)	0.401
	MTT–SMTT	0.4 (−3.5, 4.2)	0.844

*CI* confidence interval, *CRAE* central retinal arteriolar equivalent, *CRVE* central retinal venular equivalent, *MTT* maximal treadmill test, *SMTT* submaximal treadmill test, *CC* control condition.

^a^Available in 16 (94 %) and 15 (100 %) seniors and young adults, respectively

On the other hand, the dilatation in CRAE and CRVE after MTT relative to CC was more pronounced in seniors compared with young adults at t_5_, with an estimated difference of 2.9 μm (95 % CI −0.5, 6.3; *P* = 0.088) for CRAE and of 5.0 μm (95 % CI 1.5, 8.4; *P* = 0.006) for CRVE (Fig. 3a–f; Table 4). At t_40_, there was no apparent difference between seniors and young adults in the difference between MTT and CC (CRAE 0.3 μm (95 % CI −4.2, 4.9; *P* = 0.878); CRVE 1.7 μm (95 % CI −2.3, 5.6; *P* = 0.401)).

In seniors, average CRAE and CRVE at t_5_ were slightly higher for MTT compared with SMTT; while in young adults, both parameters were, on average, lower for MTT compared with SMTT. The estimated difference between seniors and young adults in the difference between MTT and SMTT was 5.3 μm (95 % CI 2.0, 8.5; *P* = 0.002) for CRAE and 4.1 μm (95 % CI −0.4, 8.6; *P* = 0.076) for CRVE (Fig. 3a–f; Table 4). At t_40_, there was no apparent difference between seniors and young adults in the difference between MTT and SMTT (CRAE 1.4 μm (95 % CI −2.3, 5.1; *P* = 0.438); CRVE 0.4 μm (95 % CI −3.5, 4.2; *P* = 0.844)).

## Discussion

The effect of endurance exercise on the systemic circulation is determined by an increase of cardiac output, rise in systolic arterial blood pressure and pulse rate as well as a decrease in peripheral vascular resistance. The retinal microcirculation, however, can maintain a constant blood flow despite a constant increase in blood pressure during exercise due to a vasoconstriction of the arterioles in response to an increase in intraluminal pressure. This regulatory mechanism is called myogenic response, also known as Bayliss effect. The myogenic autoregulation in the retinal microcirculation has been described using different imaging techniques, most of which involve considerable technical complexity and difficulties (Dumskyj et al. 1996; Jeppesen et al. 2004). This study, for the first time, used the simple approach of non-invasive and non-mydriatic static retinal vessel analysis to investigate the effect of exercise intensity on retinal vessel diameter regulation and whether this effect is age dependent.

Our results suggest an increase in retinal arteriolar and venular diameter 5 min after exercise cessation in all participants. These findings are in line with previous findings of dilated retinal vessels at delayed time points after dynamic exercise (Pressler et al. 2011; Rueddel et al. 2012). Higher intensities (MTT) seem to induce longer-lasting effects on arteriolar and venular retinal dilatation with persistent diameter increases 40 min after maximal exhaustive exercise. Dilated retinal vessel diameters after exercise seem to stand in contrast to the above described physiological vasoconstriction of retinal vessels during exercise. How can this “paradox” be explained?

Retinal arteries have unusually developed smooth muscle layers compared to other arteries with more or less negligible autonomic nerve stimulation (Pournaras et al. 2008). Retinal vessel blood flow and diameter regulation depends in large part on endothelial and microvascular smooth muscle activity. High levels of circulating catecholamines, such as after acute bouts of exercise, have been shown to have negligible effects on retinal vessel diameter, tone and blood flow (Jandrasits et al. 2002). In a simplistic model, the fine interplay between vasodilatory effects such as nitric oxide-mediated shear stress and vasoconstrictive effects such as the Bayliss effect as well as changes in blood gases (hypocapnia) and local vasoactive peptides determine the retinal vasomotor tone and, thereby, regulate retinal blood flow (Pournaras et al. 2008). During high exercise intensities, the myogenic vasoconstriction in response to increased intraluminal pressure overrides the vasodilatory effects, thereby ensuring constant retinal blood flow. The myogenic response is further aggravated by the hyperventilation-induced hypocapnia during higher and maximal exercise. Low arterial partial pressures of CO_2_ (PaCO_2_) induce retinal vasoconstriction (Pournaras et al. 2008; Ikemura and Hayashi 2012). As the haemodynamic and metabolic stimuli normalize in the recovery phase, vasodilatory mechanisms become predominant, ensuring a steady blood flow after cessation of exercise.

Our results indicate that age and exercise intensity affect the regulation of retinal vascular diameters, which account for changes in blood flow and vascular resistance. Blood flow in retinal arterioles of healthy middle-aged individuals has previously been shown to be increased during submaximal exercise, whereas it is reduced during maximal exercise (Ikemura and Hayashi 2012). These findings of cerebrovascular blood flow patterns in response to exercise correspond with our findings of retinal diameter changes in response to exercise in healthy young individuals.

In our study, a walking-based submaximal exercise induced a dilatation of retinal arterioles 5 and 40 min after exercise, which was more pronounced in healthy young adults. This finding can be explained by the fact that endothelial function deteriorates with increasing age with the dilatory properties gradually declining (Hatake et al. 1990; Mayhan et al. 2008). At high exercise intensities, the myogenic properties become the key regulative mechanism in response to increases in intraluminal pressure. The physiological autoregulated myogenic vasoconstriction after MTT is the most likely an explanation for the narrower retinal arterioles after MTT compared to the submaximal walking exercise (Fig. 3c) at younger age. In young adults, our findings are in line with the physiological principles of a normal vascular reactivity. In seniors, MTT does not induce significant lower retinal arteriolar diameters compared to SMTT (Fig. 3c). These findings can be interpreted as a loss of vascular reactivity in response to high exercise intensities in seniors relative to young adults, affecting the myogenic properties of the vessels. Differences in PetCO_2_ and estimated PaCO_2_ in seniors do not account for this impairment (Table 2). Marsden et al. have recently examined the effect of age on middle cerebral artery blood flow in response to increasing intensities of exercise (Marsden et al. 2012). They found a reduction of cerebral blood flow elevation at lower intensities and a blunted cerebral blood flow reduction at higher exercise levels (>70 % peak oxygen uptake) in seniors compared to the young.

Our results of a decreased myogenic response in the elderly are based on findings of diameter changes rather than alterations in cerebral blood flow, but they are in line with these previous findings. Autoregulated changes in diameter account for changes in blood flow. Our study, for the first time, demonstrates that the relatively simple measurement of retinal vessel diameters, a valid new tissue biomarker of cardiovascular risk, corresponds to previously described cerebral artery blood flow alterations in response to exercise. By use of retinal vessel diameters, a reduced dilatation in response to low exercise intensities and an impaired myogenic vasoconstriction at higher exercise intensities can be differentiated in seniors compared to young adults. The vasoconstrictive counterregulation of retinal arterioles and venules in the young almost disappeared after 40 min. We know from several large cohort studies that retinal vessel diameters can be used as tissue biomarkers of cardiovascular risk prediction in cross-sectional approaches, as described in the Introduction. This study shows that retinal vessel diameters can also depict age-dependent decrease in vascular reactivity in the setting of acute exercise interventions.

The reduction of the myogenic response in seniors is of high clinical relevance. In contrast to a decrease of peripheral vascular resistance, cerebrovascular resistance increases by about 40 % after exercise (Globus et al. 1983). The metabolic and haemodynamic changes in response to higher exercise intensities lead to an increase of total cerebrovascular resistance, which is achieved by myogenic vasoconstriction preventing a rise of cerebrovascular blood flow during and after exercise. The counterregulative myogenic response seems to be protective, preventing an overshooting of flow to the brain and, potentially, haemorrhage.

Interestingly, our findings of retinal arteriolar diameter changes in response to exercise are very similar to those of venular diameter changes. The age-dependent loss of vasoconstrictive properties of cerebral veins has not been described before. Since the venous response to exercise in the young and old seems to correspond with the arterial pattern, it may be assumed that similar haemodynamic and metabolic mechanisms are responsible for the diameter changes in venules. However, retinal veins are more passively regulated and, since inflammatory cytokines are known to influence retinal venular diameter, release of cytokines such as interleukin-6 in response to exhaustive exercise may also affect venular diameters (Wong et al. 2006a; Klein et al. 2006).

Our study presents some limitations. The retinal images were taken at baseline as well as 5 and 40 min after exercise and could not be monitored continuously. However, the postmeasurements were standardized at fixed postexercise times and are, therefore, inter- and intraindividually comparable. The control group was resting in supine position and had to change into sitting position for retinal vessel imaging. Orthostatic changes during this manoeuvre may have led to a slight overestimation of the exercise effects over control. However, our main finding of a reduced vascular reactivity in seniors relative to young adults after maximal compared to submaximal exercise does not depend on the control condition. A cross-over design in which each study participant is given each exercise mode and the control condition just once may not be ideal to assess whether the effects of exercise differ between seniors and young adults. It does however allow us to study the differences in the way in which seniors and young adults respond to exercise.

Exploring the molecular mechanisms underlying the vascular reactivity in the young and old, such as nitric oxide bioavailability, was beyond the scope of the study. We solely relied on the measurement of retinal vessel diameters to analyse vascular reactivity in response to exercise. Retinal vessel analysis has gained considerable interest in the last few years as an emerging vascular biomarker of the cerebral microcirculation. Diameter changes regulate blood flow and the accordance with previous findings of cerebral blood flow in response to exercise nicely demonstrates the physiological role of retinal vessel diameter changes.

## Conclusions

Using static retinal vessel diameter analysis, our results suggest an impairment of myogenic vasoconstriction with older age and in response to higher exercise intensities in arterioles as well as venules. Normal vascular reactivity ensures an intact autoregulation of the retinal vasculature. The loss of myogenic vasoconstriction and the associated inability to increase vascular resistance in response to intraluminal pressure increase after exercise imply an increased risk of retinal and potentially cerebral haemorrhage in the elderly. Future studies need to investigate whether regular endurance exercise has the potential to improve cerebrovascular reactivity in the elderly and whether haemodynamic adaptations to chronic exercise correspond to improvements in cognitive function and reduced risk of stroke in the clinical setting.

## Electronic supplementary material

Below is the link to the electronic supplementary material.

ESM 1 (DOC 46 kb)
