# Supplementary material for: Effects of acute bouts of endurance exercise on retinal vessel diameters are age and intensity dependent
Source: Age (Dordr). 2014 Apr 12;36(3):9650. doi: 10.1007/s11357-014-9650-3 (PMC4082577; doi:10.1007/s11357-014-9650-3)
Supplement: Supplementary file 1 — (DOC 46 kb) [file 11357_2014_9650_MOESM1_ESM.doc]

**Supplement data:** Blood pressure at baseline, as well as 5 and 40 minutes after each exercise mode, with data summarised as median (interquartile range).

|  | baseline | t_5_ | t_40_ |
| --- | --- | --- | --- |
| ***Maximum treadmill test (MTT)*** | | | |
| *Systolic blood pressure (SBP) in mmHg* | | | |
| All participants | 124 (115, 137) | 114 (107, 130) | 112 (105, 121) |
| Seniors | 133 (124, 139)^*^ | 130 (114, 136)^#^ | 120 (106, 126)^*^ |
| Young adults | 115 (108, 124) | 113 (103, 115) | 110 (102, 115) |
| *Diastolic blood pressure (DBP)in mmHg* | | | |
| All participants | 80 (74, 88) | 77 (73, 82) | 76 (70, 81) |
| Seniors | 85 (80, 88)^*^ | 79 (74, 82)^#^ | 78 (71, 82)^*^ |
| Young adults | 78 (73, 81) | 76 (73, 81) | 73 (69, 78) |
| ***Submaximal 2-km treadmill test (SMTT)*** | | | |
| *Systolic blood pressure (SBP) in mmHg* | | | |
| All participants | 120 (105, 132) | 114 (108, 128) | 117 (105, 122) |
| Seniors | 127 (107, 137) | 124 (112, 130) | 118 (106, 128) |
| Young adults | 114 (104, 124) | 110 (107, 120) | 109 (105, 120) |
| *Diastolic blood pressure (DBP)in mmHg* | | | |
| All participants | 77 (70, 83) | 76 (70, 82) | 74 (68, 78) |
| Seniors | 77 (71, 83) | 78 (71, 81) | 77 (69, 79) |
| Young adults | 72 (70, 78) | 74 (70, 83) | 74 (71, 76) |
| ***Control condition (CC)*** | | | |
| *Systolic blood pressure (SBP) in mmHg* | | | |
| All participants | 120 (107, 125) | 123 (114, 113) | 124 (111, 130) |
| Seniors | 123 (117, 136)^*^ | 133 (116, 143)^*^ | 128 (114, 136)^*^ |
| Young adults | 112 (106, 123) | 119 (111, 124) | 117 (107, 126) |
| *Diastolic blood pressure (DBP)in mmHg* | | | |
| All participants | 74 (72, 76) | 78 (71, 83) | 78 (71, 82) |
| Seniors | 74 (71, 79)^*^ | 79 (70, 86)^*^ | 78 (70, 85)^*^ |
| Young adults | 75 (72, 76) | 75 (72, 81) | 78 (71, 81) |
| ^*^Available in 14 (82%) seniors  ^#^Available in 13 (76%) seniors | | | |
